# Hemostats in the clinic

**DOI:** 10.1002/btm2.10673

**Published:** 2024-05-01

**Authors:** Maithili Joshi, Zongmin Zhao, Samir Mitragotri

**Affiliations:** ^1^ John A. Paulson School of Engineering and Applied Sciences Harvard University Allston Massachusetts USA; ^2^ Wyss Institute for Biologically Inspired Engineering at Harvard University Boston Massachusetts USA; ^3^ Department of Pharmaceutical Sciences, College of Pharmacy University of Illinois at Chicago Chicago Illinois USA

**Keywords:** clinic, clinical translation, clinical trial, coagulation, FDA, hemophilia, hemorrhage, hemostat, trauma

## Abstract

Given the prevalence of hematological conditions, surgeries, and trauma incidents, hemostats—therapeutics designed to control and arrest bleeding—are an important tool in patient care. The prophylactic and therapeutic use of hemostats markedly enhances survival rates and improves the overall quality of life of patients suffering from these conditions. Since their inception in the 1960s, hemostats have witnessed remarkable progress in terms of the active ingredients utilized, therapeutic outcomes, demonstrated efficacy, and the storage stability. In this review, we provide a comprehensive analysis of commercially available hemostats approved by the FDA, along with newer investigative hemostats currently in active clinical trials. We delve into the modality of active ingredients, route of administration, formulation type, and disease indications of these approved and investigative hemostats. Further, we analyze the trends observed in the hemostat actives for Hemophilia A and B, concluding with insights into the emerging patterns and noteworthy developments to watch for in this dynamic field.

**Translational Impact Statement**

Over the past six decades, the field of hemostats has undergone remarkable advancements, evident in the evolution of many approved products encompassing diverse drug classes. The recent shift toward the use of gene therapy agents and the ongoing progress in the development of protein modalities open new possibilities for the advancement of novel hemostats, addressing previously unsolved challenges in the realms of hemorrhage and internal bleeding. This article offers a comprehensive review of the history and trajectory of hemostats, emphasizing the substantial translational impact achieved in the field of hemostasis, while acknowledging persisting challenges. Moving forward, the dynamic and evolving landscape of this essential class of agents is highlighted by emerging trends and the ongoing pursuit of innovative hemostat formulations.

## 1 INTRODUCTION

Trauma, surgery and coagulation factor deficiencies constitute the primary causes of bleeding incidences. Traumatic injuries, marked by their rapid onset and high severity, disrupt hemostasis—the body's normal physiological response to prevent blood loss—rendering it ineffective and insufficient. One of the most severe consequences of such injuries is exsanguination, leading to low blood volume and the potential for fatal complications. This blood volume depletion results in diminished oxygen delivery to the microcirculation, culminating in the lethal triad of coagulopathy, hypothermia, and acidosis.^1^, ^2^, ^3^ Such bleeding (or hemorrhage) associated with trauma is a primary cause of death, accounting for 43% and 90% of deaths in the civilian and military settings, respectively.^2^, ^4^, ^5^ Survival after injury is intricately tied to the time from injury to hemostatic intervention, emphasizing the critical importance of the “golden hour” in trauma care. Timely hemostatic intervention within the initial hour significantly determines the survival of trauma patients.^1^, ^6^ In addition, complex surgeries often involve profuse hemorrhage, amplifying morbidity and mortality risks. Bleeding complications during operative procedures can lead to hemodilution, hypothermia, clotting factor depletion, and acidosis.^7^, ^8^ In cases of internal bleeding, early intervention is extremely critical; however, effective agents for bleeding control are currently lacking.^9^, ^10^, ^11^ Moreover, bleeding disorders, which are characterized by the deficiency of one or more factors involved in one of the stages of hemostasis, result in coagulopathy and an inability of the body to form a clot, resulting in excessive bleeding. The prevalent coagulation factor deficiencies manifest in three main conditions: Hemophilia A (Factor VIII deficiency), Hemophilia B (Factor IX deficiency), and von Willebrand disease (deficient or defective plasma von Willebrand Factor).^12^, ^13^ In addition to these, recent discoveries of new clotting disorders have shed light on previously unexplored aspects of coagulation abnormalities.^12^

Bleeding due to these conditions—trauma, surgery and coagulation factor deficiencies, necessitates the administration of specific agents, known as hemostats, to prevent and treat life‐threatening bleeding episodes. Hemostats that can achieve prompt and effective control of bleeding are instrumental agents in the prevention of death due to hemorrhage. Over the past 60 years, there has been a striking progress in the field of hemostats in terms of development of novel active ingredients, prophylactic effectiveness and therapeutic adherence.^13^ This is reflected in the increasing number of hemostats being approved by the US Food and Drug Administration (FDA) per decade. In this review, we provide a comprehensive review of hemostats that are approved by the FDA or are currently in active clinical trials. Agents that directly take part in primary/secondary hemostasis or directly activate a component participating in hemostasis or prevent pathways that inhibit clot formation (e.g., fibrinolysis) are included in our discussions. Agents that do not directly participate in the physiological process of hemostasis but rather activate pathways/receptors/proteins that eventually activate/produce a component involved in hemostasis are not included in this review. In addition, products that are approved or under investigation as medical devices (e.g., QuikClot, Surgicel, XStat, etc.) are not included in our discussion. More information about these medical device‐based hemostats can be found in other reviews published elsewhere.^14^, ^15^, ^16^ We also specifically discuss the evolution of hemostat agents used for managing Hemophilia A and B and provide insights on the emerging trends and future directions in the field of hemostats in the forthcoming years.

## 2 HEMOSTASIS: PHYSIOLOGY AND COMPONENTS

**FIGURE 1:**
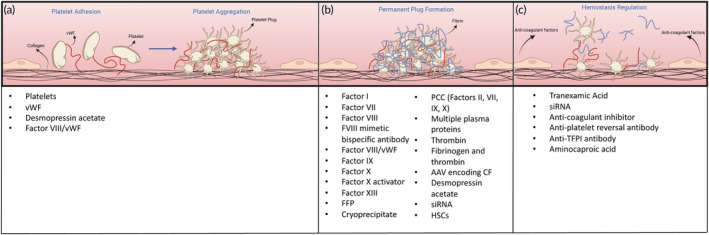
Stages of hemostasis and corresponding agents used to enhance the respective stage. (a) Primary hemostasis involves platelet adhesion and platelet aggregation which eventually leads to platelet plug formation. (b) Secondary hemostasis involves the coagulation cascade which terminates with the formation of a permanent plug characterized by a fibrin mesh. (c) Regulation of hemostasis consists of a delicate balance between pro‐coagulant and anti‐coagulant factors. Anti‐coagulant factors released by the endothelial cells limit hemostasis to the site of vascular injury. In cases of elevated anti‐coagulant activity, agents that inhibit anti‐coagulants are employed as hemostats. FFP, fresh frozen plasma; HSC, hematopoietic stem cell; siRNA, small interfering RNA; TFPI, tissue‐factor pathway inhibitor; vWF, von Willebrand Factor.

Hemostasis, an intricately orchestrated multi‐stage process, represents the body's innate physiological response to cease bleeding by forming a localized plug in injured vessels, while preserving regular blood flow in the broader circulation. Primary and secondary hemostasis constitute the two main components of this system, working in close coordination and occurring nearly simultaneously.^17^ Figure 1 shows the steps involved in hemostasis and the corresponding hemostatic agents (clinically used or in clinical trials) that enhance those steps.

Platelets are the principal cell type involved in primary hemostasis. Platelets are anucleate cell fragments that arise from the budding of larger megakaryocytes in the bone marrow.^17^ During primary hemostasis, platelets adhere to the site of the injury and subsequently aggregate to form a platelet plug (Figure 1a). This is mediated by platelet receptors and adhesive proteins derived from platelets and plasma.^18^, ^19^ Of note is the von‐Willebrand factor (vWF), a large multimeric protein secreted by both platelets and endothelial cells. Upon activation, vWF binds to exposed collagen at the injury site and the platelet receptor GPIbα, thus enhancing platelet adhesion at the injury site.^17^ Platelet deficiency resulting from significant blood loss due to hemorrhage, surgery, or underlying bleeding disorders requires extensive transfusions to elevate the platelet count to a level conducive for adequate hemostasis.^18^ Deficiency of vWF leads to von Willebrand disease, the most common bleeding disorder that causes delays in clot formation.^19^, ^20^ Thus, agents that enhance or augment primary hemostasis include platelets, vWF, desmopressin acetate (increases vWF secretion and coagulation factor VIII activity)^21^ and the Factor VIII/vWF complex. A detailed description of these agents is provided in Sections 3 and 4.

Primary hemostasis is followed by secondary hemostasis, engaging the coagulation cascade. Several different coagulation proteins are involved in this cascade that eventually culminates in the formation of an insoluble fibrin mesh that gets incorporated into the platelet plug.^22^ Of these, Factors VII, VIII and IX are studied most extensively in the context of clotting disorders and replacement therapies. The coagulation cascade is activated by two pathways, the tissue factor pathway (extrinsic pathway) and the contact activation pathway (intrinsic pathway). The activation of both pathways ultimately results in the activation of prothrombin to thrombin, leading to the conversion of fibrinogen to fibrin to form a stable clot.^23^ Severe injury and massive blood loss during surgery can lead to a rapid deficiency of clotting factors. Such conditions require the administration of a single or a combination of clotting factors to ensure secondary hemostasis occurs. In addition, congenital and acquired coagulation factor deficiencies also necessitate the administration of the required coagulation factor as a replacement therapy or bypassing agent.^24^, ^25^ Agents that can restore impaired secondary hemostasis are listed in Figure 1b and are also discussed in detail in later sections.

In addition to the two stages of hemostasis, fibrinolysis is an equally important process that dissolves blood clots no longer required, in order to keep healthy blood vessels open for unrestricted circulation. Agents involved in this system include anti‐coagulant factors such as prostacyclin (PGI), anti‐thrombin III, proteins C and S, tissue factor pathway inhibitor (TFPI), and tissue‐type plasminogen activator (t‐PA).^26^ However, an impaired fibrinolysis system may lead to the premature dissolution of the blood clot, thus leading to bleeding. As a result, agents that counter anti‐coagulant factors and help maintain the integrity of the permanent plug are also used as hemostats, as shown in Figure 1c. Several reviews published elsewhere discuss the processes of primary and secondary hemostasis and fibrinolysis in greater detail.^22^, ^23^, ^27^, ^28^, ^29^

## 3 FDA‐APPROVED HEMOSTATS

A total of 54 hemostats have been approved by the FDA to date (Table 1), as either new drug applications (NDAs) or biologics license applications (BLAs). These hemostats span a wide array of active ingredients (16 unique active ingredients), indications, modalities, formulation types and routes of administration. Here we provide an overview of these approved hemostats and discuss active clinical trials investigating these approved hemostats for new indications or demographics.

**TABLE 1:** FDA approved commercially available hemostats.

Trade name	Company	Active ingredient (class^a^)	STN (approval year)	Formulation	Route (half‐life^a^)	Frequency of administration (prophylactic dosing^a^)	Indication	Hemorrhage specifics	Warnings
Proteins									
HEMLIBRA®	Genentech	Bispecific factor IXa‐ & factor X‐directed antibody (Monoclonal Antibody)	BLA 761083 (2017)	Solution	SC (4 weeks)	Every week, every 2 weeks, or every 4 weeks	Hemophilia A	Routine prophylaxis to prevent or reduce the frequency of bleeding episodes	Thrombotic microangiopathy and thromboembolism when given with aPCC
Profilnine®	Grifols	CF II, VII, IX and X (PCC)	BLA 102476 (1981)	Lyophilized Powder for Solution	IV (24 h)	Every 16–24 h, for 1–10 days	Hemophilia B	Prevention and control of bleeding in patients	Thrombosis or DIC
FEIBA®	Takeda	CF II, VII, IX and X (PCC)	BLA 101447 (1986)	Lyophilized Powder for Solution	IV	Every 6–12 h; (every other day)	Hemophilia A and B	Control and prevention of bleeding, perioperative management, prophylaxis	Thromboembolic events
Kcentra®	CSL Behring	CF II, VII, IX, X, Proteins C & S (PCC)	BLA 125421 (2013)	Lyophilized Powder for Solution	IV	During need for urgent surgery/acute major bleeding	Acquired CF deficiency induced by Vitamin K antagonist	Acute Major Bleeding/surgery/invasive procedure	Hypersensitivity reactions, thromboembolic risk/complications, transmissible infectious agents
Balfaxar®	Octapharma	CF II, VII, IX, X, Proteins C & S (PCC)	BLA 125776 (2023)	Lyophilized Powder for Solution	IV	During need for urgent surgery/acute major bleeding	Acquired CF deficiency induced by Vitamin K antagonist	Acute Major Bleeding/surgery/invasive procedure	Hypersensitivity reactions, thromboembolic risk/complications, transmissible infectious agents
RiaSTAP®	CSL Behring	FI	BLA 125317 (2009)	Lyophilized Powder for Solution	IV (78.7 h)	Based on extent of bleeding and clinical condition	Congenital fibrinogen deficiency, including afibrinogenemia and hypofibrinogenemia	Acute bleeding episodes in pediatric and adult patients with congenital fibrinogen deficiency, including afibrinogenemia and hypofibrinogenemia	Hypersensitivity reactions, thrombosis, transmissible infectious agents
Fibryga®	Octapharma	FI	BLA 125612 (2017)	Lyophilized Powder for Solution	IV (75.9 h)	Based on extent of bleeding and clinical condition	Congenital fibrinogen deficiency, including afibrinogenemia and hypofibrinogenemia	Acute bleeding episodes in adults and adolescents with congenital fibrinogen deficiency, including afibrinogenemia and hypofibrinogenemia.	Hypersensitivity reactions, thrombosis, transmissible infectious agents
NovoSeven®	Novo Nordisk	FVII	BLA 103665 (1999)	Lyophilized Powder for Solution	IV (2.3 h)	Every 2–3 h until hemostasis achieved (Hemophilia), every 4–6 h (Congenital FVII deficiency)	Hemophilia A or B, congenital FVII deficiency	Treatment of bleeding episodes, prevention of bleeding in surgical interventions or invasive procedures	Thrombotic events
SevenFACT®	Hema Biologics	FVII	BLA 125641 (2020)	Lyophilized Powder for Solution	IV (1.6 h)	Every 3 h until hemostasis achieved	Hemophilia A or B	Treatment and control of bleeding episodes	Thrombosis, hypersensitivity reactions, neutralizing antibodies
HemofilM®	Takeda	FVIII	BLA 101448 (1966)	Lyophilized Powder for Solution	IV (14.8 h)	Every 12–24 h until healing achieved	Hemophilia A	Prevention and control of hemorrhagic episodes	Hypersensitivity, neutralizing antibodies, transmission of infectious agents
KOATE®	Kedrion BioPharma	FVIII	BLA 101130 (1974)	Lyophilized Powder for Solution	IV (16.1 h)	Every 12 h until healing achieved	Hemophilia A	Control and prevention of bleeding episodes or in order to perform emergency and elective surgery	Hypersensitivity reactions, neutralizing antibodies, intravascular hemolysis, transmissible infectious agents
Recombinate™	Takeda	FVIII	BLA 103375 (1992)	Lyophilized Powder for Solution	IV (14.7 h)	Every 12–24 h until healing achieved	Hemophilia A	Prevention and control of hemorrhagic episodes, perioperative management of patients	Anaphylaxis and severe hypersensitivity reactions, neutralizing antibodies
ReFacto®	Pfizer	FVIII	BLA 103779 (2000)	Lyophilized Powder for Solution	IV (13.7 h)	Every 12–24 h until healing achieved (2–3 times weekly)	Hemophilia A	Control and prevention of hemorrhagic episodes and for surgical prophylaxis in patients; short‐term routine prophylaxis to reduce the frequency of spontaneous bleeding episodes.	Hypersensitivity reactions, neutralizing antibodies
ADVATE®	Takeda	FVIII	BLA 125063 (2003)	Lyophilized Powder for Solution	IV (12.3 h)	Every 12–24 h until healing achieved (3–4 times weekly)	Hemophilia A	(1) Routine prophylaxis to reduce the frequency of bleeding episodes; (2) On‐demand treatment and control of bleeding episodes; and (3) Perioperative management of bleeding.	Anaphylaxis and hypersensitivity reactions, neutralizing antibodies
XYNTHA®	Pfizer	FVIII	BLA 125264 (2008)	Lyophilized Powder for Solution	IV (11.2 h)	Every 12–24 h until healing achieved (3 times weekly)	Hemophilia A	(1) Routine prophylaxis to reduce the frequency of bleeding episodes; (2) On‐demand treatment and control of bleeding episodes; and (3) Perioperative management of bleeding.	Hypersensitivity reactions, neutralizing antibodies
Novoeight®	Novo Nordisk	FVIII	BLA 125466 (2013)	Lyophilized Powder for Solution	IV (10.8 h)	Every 12–24 h until healing achieved (3 times weekly)	Hemophilia A	(1) Routine prophylaxis to reduce the frequency of bleeding episodes; (2) On‐demand treatment and control of bleeding episodes; and (3) Perioperative management of bleeding.	Hypersensitivity reactions, neutralizing antibodies
Obizur®	Takeda	FVIII	BLA 125512 (2014)	Lyophilized Powder for Solution	IV (N/A)	Every 4–12 h	Hemophilia A	On‐demand treatment and control of bleeding episodes in adults with acquired Hemophilia A	Hypersensitivity reactions, neutralizing antibodies
ELOCTATE®	Sanofi	FVIII‐Fc Fusion Protein	BLA 125487 (2014)	Lyophilized Powder for Solution	IV (19.7 h)	Every 24–28 h until healing achieved (every 4 days)	Hemophilia A	(1) Routine prophylaxis to reduce the frequency of bleeding episodes; (2) On‐demand treatment and control of bleeding episodes; and (3) Perioperative management of bleeding.	Hypersensitivity reactions, neutralizing antibodies
NUWIQ®	Octapharma	FVIII	BLA 125555 (2015)	Lyophilized Powder for Solution	IV (17.1 h)	Every 12–24 h until healing achieved (every other day)	Hemophilia A	(1) Routine prophylaxis to reduce the frequency of bleeding episodes; (2) On‐demand treatment and control of bleeding episodes; and (3) Perioperative management of bleeding.	Hypersensitivity reactions, neutralizing antibodies
ADYNOVATE®	Takeda	PEGylated FVIII	BLA 125566 (2015)	Lyophilized Powder for Solution	IV (14.7 h)	Every 12–24 h until healing achieved (twice weekly)	Hemophilia A	(1) Routine prophylaxis to reduce the frequency of bleeding episodes; (2) On‐demand treatment and control of bleeding episodes; and (3) Perioperative management of bleeding.	Hypersensitivity reactions, neutralizing antibodies
KOVALTRY®	Bayer	FVIII	BLA 125574 (2016)	Lyophilized Powder for Solution	IV (14.3 h)	Every 12–24 h until healing achieved (2–3 times weekly)	Hemophilia A	(1) Routine prophylaxis to reduce the frequency of bleeding episodes; (2) On‐demand treatment and control of bleeding episodes; and (3) Perioperative management of bleeding.	Hypersensitivity reactions, neutralizing antibodies
AFSTYLA®	CSL Behring	FVIII‐sc	BLA 125591 (2016)	Lyophilized Powder for Solution	IV (14.2 h)	Every 12–24 h until healing achieved (2–3 times weekly)	Hemophilia A	(1) Routine prophylaxis to reduce the frequency of bleeding episodes; (2) On‐demand treatment and control of bleeding episodes; and (3) Perioperative management of bleeding.	Hypersensitivity reactions, neutralizing antibodies
JIVI®	Bayer	PEGylated FVIII	BLA 125661 (2018)	Lyophilized Powder for Solution	IV (18.6 h)	Every 24–48 h until healing achieved (2 times weekly)	Hemophilia A	(1) Routine prophylaxis to reduce the frequency of bleeding episodes; (2) On‐demand treatment and control of bleeding episodes; and (3) Perioperative management of bleeding.	Hypersensitivity reactions, FVIII neutralizing antibodies, immune response to PEG
Esperoct®	Novo Nordisk	GlycoPEGylated FVIII	BLA 125671 (2019)	Lyophilized Powder for Solution	IV (21.7 h)	One dose generally sufficient (every 4 days)	Hemophilia A	(1) Routine prophylaxis to reduce the frequency of bleeding episodes; (2) On‐demand treatment and control of bleeding episodes; and (3) Perioperative management of bleeding.	Hypersensitivity reactions, FVIII neutralizing antibodies
ALTUVIIIO®	Sanofi	FVIII‐Fc‐VWF‐(XTEN)2 fusion protein	BLA 125771 (2023)	Lyophilized Powder for Solution	IV (48.2 h)	Single dose (once weekly)	Hemophilia A	(1) Routine prophylaxis to reduce the frequency of bleeding episodes; (2) On‐demand treatment and control of bleeding episodes; and (3) Perioperative management of bleeding.	Hypersensitivity reactions, FVIII neutralizing antibodies
Alphanate®	Grifols	FVIII/vWF	BLA 102475 (1978)	Lyophilized Powder for Solution	IV (17.9 h [FVIII in Hemophilia A], 7.7 h [vWF:Rco in vWD], 21.6 h [FVIII in vWD])	Every 8 to 12 h as clinically needed	Hemophilia A, vWD	(1) Control and prevention of bleeding in patients with Hemophilia A or acquired Factor VIII (FVIII) deficiency, (2) Surgical and/or invasive procedures in adult and pediatric patients with von Willebrand Disease in whom desmopressin (DDAVP) is contraindicated	Hypersensitivity reactions, neutralizing antibodies, thromboembolic events, intravascular hemolysis, vasomotor reactions, transmissible infectious agents
HUMATE‐P®	CSL Behring	FVIII/vWF	BLA 103960 (1986)	Lyophilized Powder for Solution	IV (12.2 h [FVIII in Hemophilia A], 11 h [vWF: Rco in vWD])	Once/twice a day as clinically needed	Hemophilia A, vWD	(1) Treatment of spontaneous and trauma‐induced bleeding episodes, and (2) Prevention of excessive bleeding during and after surgery.	Thromboembolic events, transmissible infectious agents
Wilate®	Octapharma	FVIII/vWF	BLA 125251 (2009)	Lyophilized Powder for Solution	IV (10.6 h [FVIII in Hemophilia A], 17.5 h [FVIII in vWD]), 24.7 h [vWF: Rco in vWD I])	Every 12–24 h as clinically needed (2–3 times weekly)	Hemophilia A, vWD	Hemophilia: Routine prophylaxis to reduce the frequency of bleeding episodes, On‐demand treatment and control of bleeding episodes; vWD: On‐demand treatment and control of bleeding episodes, Perioperative management of bleeding	Hypersensitivity reactions, thromboembolic events, FVIII neutralizing antibodies, transmissible infectious agents
AlphaNine®	Grifols	FIX	BLA 103249 (1996)	Lyophilized Powder for Solution	IV (21 h)	Twice daily until healing achieved	Hemophilia B	Prevention and control of bleeding	Transmissible infectious agents, thrombosis, hypersensitivity reactions, nephrotic syndrome
BeneFix®	Pfizer	FIX	BLA 103677 (1997)	Lyophilized Powder for Solution	IV (22.4 h)	Every 12–24 h as clinically needed (once weekly)	Hemophilia B	(1) On‐demand treatment and control of bleeding episodes, (2) Perioperative management of bleeding, (3) Routine prophylaxis to reduce the frequency of bleeding episodes.	Thromboembolic complications, hypersensitivity reactions, nephrotic syndrome, FIX neutralizing antibodies
RIXUBIS®	Takeda	FIX	BLA 125446 (2013)	Lyophilized Powder for Solution	IV (26.7 h)	Every 12–24 h as clinically needed (twice weekly)	Hemophilia B	Control and prevention of bleeding episodes, perioperative management, and routine prophylaxis	Thromboembolic complications, hypersensitivity reactions, nephrotic syndrome, FIX neutralizing antibodies
ALPROLIX®	Sanofi	FIX‐Fc Fusion protein	BLA 125444 (2014)	Lyophilized Powder for Solution	IV (86 h)	Every 48 h until healing achieved (once every 10 days)	Hemophilia B	(1) On‐demand treatment and control of bleeding episodes, (2) Perioperative management of bleeding, (3) Routine prophylaxis to reduce the frequency of bleeding episodes.	Thromboembolic complications, hypersensitivity reactions, nephrotic syndrome, FIX neutralizing antibodies
IXINITY®	Medexus Pharma	FIX	BLA 125426 (2015)	Lyophilized Powder for Solution	IV (24 h)	Every 24 h until healing achieved (twice weekly)	Hemophilia B	(1) On‐demand treatment and control of bleeding episodes, (2) Perioperative management of bleeding, (3) Routine prophylaxis to reduce the frequency of bleeding episodes.	Thromboembolic complications, hypersensitivity reactions, nephrotic syndrome, FIX neutralizing antibodies
IDELVION®	CSL Behring	Albumin FP‐FIX	BLA 125582 (2016)	Lyophilized Powder for Solution	IV (104 h)	Every 48–72 h until healing achieved (once weekly)	Hemophilia B	(1) On‐demand treatment and control of bleeding episodes, (2) Perioperative management of bleeding, (3) Routine prophylaxis to reduce the frequency of bleeding episodes.	Thromboembolic complications, hypersensitivity reactions, nephrotic syndrome, FIX neutralizing antibodies
Rebinyn®	Novo Nordisk	GlycoPEGylated FIX	BLA 125611 (2017)	Lyophilized Powder for Solution	IV (114.9 h)	Single dose sufficient	Hemophilia B	(1) On‐demand treatment and control of bleeding episodes, (2) Perioperative management of bleeding	Thromboembolic complications, hypersensitivity reactions, nephrotic syndrome, FIX neutralizing antibodies
COAGADEX®	Kedrion BioPharma	FX	BLA 125506 (2015)	Lyophilized Powder for Solution	IV (30.3 h)	Every 24 h until healing achieved, (twice weekly)	Hereditary Factor X deficiency	(1) Routine prophylaxis to reduce the frequency of bleeding episodes; (2) On‐demand treatment and control of bleeding episodes; and (3) Perioperative management of bleeding in those with Factor X deficiency	Hypersensitivity reactions, neutralizing antibodies, transmissible infectious agents
CORIFACT®	CSL Behring	FXIII	BLA 125385 (2011)	Lyophilized Powder for Solution	IV (6.6 days)	(Every 28 days)	Congenital Factor XIII deficiency	(1) Routine prophylactic treatment, (2) Perioperative management of surgical bleeding	Hypersensitivity reactions, immunogenicity, thromboembolic risk, transmissible infectious agents
Tretten®	Novo Nordisk	FXIII A‐Subunit	BLA 125398 (2013)	Lyophilized Powder for Solution	IV (5.1 days)	(Once monthly)	Congenital factor XIII A‐subunit deficiency	Routine prophylaxis for bleeding	Hypersensitivity reactions, thromboembolic risk, neutralizing antibodies
TISSEEL™	Baxter	Fibrinogen & thrombin (Fibrin Sealant)	BLA 103980 (1998)	Frozen solution & lyophilized powder	Topical	During surgery	Surgery	Adjunct to hemostasis in adult and pediatric patients (>1 month of age) undergoing surgery when control by conventional surgical techniques is ineffective	Hypersensitivity, transmission of infectious agents, thromboembolic events in case of intravascular application
Evicel®	Ethicon	Fibrinogen & thrombin (Fibrin Sealant)	BLA 125010 (2003)	Frozen Solutions	Topical	During surgery	Surgery	Adjunct to hemostasis in adult and pediatric patients (>1 month of age) undergoing surgery when control by conventional surgical techniques is ineffective	Hypersensitivity, transmission of infectious agents, thromboembolic events in case of intravascular application
TachoSil®	Corza Medical	Fibrinogen & thrombin (Fibrin Sealant)	BLA 125351 (2010)	Absorbable patch	Topical	During surgery	Surgery	Adjunct to hemostasis in adult and pediatric patients (>1 month of age) undergoing surgery when control by conventional surgical techniques is ineffective	Hypersensitivity, transmission of infectious agents, thromboembolic events in case of intravascular application
EVARREST®	Ethicon	Fibrinogen & thrombin (Fibrin Sealant)	BLA 125392 (2012)	Flexible composite patch	Topical	During surgery	Surgery	Adjunct to hemostasis in adult patients undergoing surgery when control by conventional surgical techniques is ineffective	Hypersensitivity, transmission of infectious agents, thromboembolic events in case of intravascular application
Vistaseal™	Ethicon	Fibrinogen & thrombin (Fibrin Sealant)	BLA 125640 (2017)	Sterile frozen solution in syringes	Topical	During surgery	Surgery	Adjunct to hemostasis in adult patients undergoing surgery when control by conventional surgical techniques is ineffective	Hypersensitivity, transmission of infectious agents, thromboembolic events in case of intravascular application
Octaplas®	Octapharma	Plasma proteins (Pooled Human Plasma)	BLA 125416 (2013)	Solution	IV	During surgery	Acquired CF deficiencies	Liver disease, patients undergoing cardiac surgery or liver transplantation	Transfusion reactions, hypervolemia, hyperfibrinolysis, thrombosis, citrate toxicity, transmission of infectious agents
THROMBIN‐JMI®	Pfizer	Thrombin	BLA 102865 (1986)	Solution	Topical	During surgery	Surgery	Oozing blood and minor bleeding when standard surgical techniques are ineffective	Hypersensitivity, thromboembolic events in case of intravascular application, inhibitory antibodies
EVITHROM™	Ethicon	Thrombin	BLA 125247 (2007)	Frozen Solution	Topical	During surgery	Surgery	Adjunct to hemostasis in adult and pediatric patients (>1 month of age) undergoing surgery when control by conventional surgical techniques is ineffective	Hypersensitivity, thromboembolic events in case of intravascular application, transmission of infectious agents
RECOTHROM®	Baxter	Thrombin	BLA 125248 (2008)	Lyophilized Powder for Solution	Topical	During surgery	Surgery	Oozing blood and minor bleeding when standard surgical techniques are ineffective	Hypersensitivity, thromboembolic events in case of intravascular application, inhibitory antibodies
VONVENDI®	Takeda	vWF	BLA 125577 (2015)	Lyophilized Powder for Solution	IV (22.6 h)	Every 8 to 24 h as clinically needed (twice weekly for prophylaxis)	vWD	(1) Routine prophylaxis to reduce the frequency of bleeding episodes; (2) On‐demand treatment and control of bleeding episodes; and (3) Perioperative management of bleeding	Embolism and thrombosis, hypersensitivity, neutralizing antibodies
Small molecules									
Amicar®	Akorn/Xanodyne	Aminocaproic acid (Fibrinolysis inhibitor)	NDA 15–197/15–230 (1964)	Solution for injection, Tablets	IV, syrup, tablet (2 h)	Injection: Administration by infusion till bleeding is controlled; Oral: Every hour till bleeding is controlled	Fibrinolytic bleeding	Surgery, aplastic anemia, hepatic cirrhosis, neoplastic diseases	Thrombosis, subendocardial hemorrhages, myocardium degeneration, muscle fiber necrosis
DDAVP®	Ferring	Desmopressin acetate (Vasopressin analog)	NDA 17–992, 19–955 (1978)	Solution	IV or SC (2.8 h)	During surgery	Hemophilia A, vWD type I	Maintain hemostasis during surgical procedures or traumatic injuries such as hemarthroses, intramuscular hematomas, or mucosal bleeding	Hypotension and hypertension, thrombosis, hypersensitivity, fluid retention
Lysteda®	Amring Pharmaceuticals	Tranexamic Acid (Antifibrinolytic)	NDA 022430 (1986)	Tablets	Oral (11 h)	2 tablets, 3 times daily for 5 days	Cyclic heavy menstrual bleeding	‐	Thrombotic events, ocular adverse effects
CYKLOKAPRON®	Pfizer	Tranexamic Acid (Antifibrinolytic)	NDA 019281 (1986)	Solution	IV (2 h)	Twice daily/once daily/every 48 h	Hemophilia (Short term use)	Reduce or prevent hemorrhage and reduce the need for replacement therapy during and following tooth extraction	Thrombosis, seizures, hypersensitivity, ocular adverse effects
AAV									
Hemgenix®	CSL Behring	AAV5 encoding FIX‐Padua (Viral Vector)	BLA 125772 (2022)	Suspension	IV	Single use IV infusion	Hemophilia B	Current or historical hemorrhage, repeated spontaneous bleeding episodes	Infusion reactions, hepatotoxicity, hepatocellular carcinogenicity
Roctavian™	BioMarin	AAV5 encoding FVIII (Viral Vector)	BLA 125720 (2023)	Suspension	IV	Single use IV infusion	Hemophilia A	One‐time gene therapy used for the treatment of adults	Infusion reactions, hepatotoxicity, thromboembolic events, hepatocellular carcinogenicity

*Note*: Half‐life mentioned is the mean half‐life in adults. Half‐life may vary depending on the dose, type of assay and patient age. Reported frequency for administration is to achieve control of bleeding in case of minor/moderate bleeding and during perioperative procedures. Frequency of administration may vary with severity of bleeding, patient age and presence of other clinical conditions. Prophylactic dosing may change based on patient's previous prophylaxis regimen and the amount of deficient factor in plasma following initial prophylaxis.

Abbreviations: AAV, adeno associated virus; aPCC, activated prothrombin complex concentrate; BLA, Biologic License Application; CF, coagulation factor; DIC, disseminated intravascular coagulation; FP, fusion protein; NDA, new drug application; sc, single chain; STN, submission tracking number; vWD, von Willebrand Disease.

^a^ Class, half‐life and prophylactic dosing mentioned if applicable.

Notably, when undergoing extensive hemorrhage, individuals may require blood transfusions to replenish the substantial volume of lost blood and simultaneously ensure an adequate supply of blood components crucial for hemostasis. This process involves transfusions of red blood cells (RBCs), platelets, cryoprecipitate, and fresh frozen plasma (FFP).^30^ RBC transfusions serve to restore the blood loss, thus ensuring sufficient oxygen‐carrying capacity for organs and microcirculation. Platelets, integral to hemostasis, are acquired through blood donations and transfused to individuals with hematological disorders or those experiencing significant hemorrhage.^18^, ^31^ Fresh frozen plasma (FFP) is derived from blood, rapidly frozen after isolation to preserve the biological activity of labile coagulation factors. Upon thawing, FFP is promptly transfused into patients.^30^, ^32^ Once thawed, FFP can also be used to prepare cryoprecipitate, which contains the cryoglobulin fraction of FFP, comprising of Factor VIII:C, von Willebrand factor (VWF), fibrinogen, fibronectin and factor XIII. Upon isolation, cryoprecipitate is refrozen in plasma and infused immediately upon thawing.^30^, ^33^ Thus, in addition to the commercial products discussed below, the transfusion of platelets, fresh frozen plasma (FFP), and cryoprecipitate also forms a critical strategy for achieving hemostasis. Although these components are not individually available as specific commercial products for distinct indications, their transfusion plays a vital role in addressing hemostatic challenges.

Damage control resuscitation is a method that achieves rapid hemorrhage control through early administration of blood products in a balanced ratio of 1:1:1 for plasma to platelets to red blood cells, a ratio akin to reconstituted whole blood. This method enables immediate correction of coagulopathy and minimizes the use of crystalloid fluids.^34^ This method was devised to correct both the early coagulopathy of trauma and the intravascular volume deficits of patients in hemorrhagic shock^35^ and has since been extensively adopted by trauma centers worldwide.^36^, ^37^

Given the growing evidence supporting balanced resuscitation as described above, the practice of using whole blood over component therapy (such as RBCs, platelets, plasma and cryoprecipitate) is reemerging, with many trauma centers returning to use low‐titer Type O Whole Blood (LTOWB).^38^, ^39^ Whole blood, originally used by the US Army until the 1960's, became less favorable due to processing and storage constraints. However, growing evidence suggesting the importance of balanced resuscitation in trauma has renewed the interest in the use of whole blood for massive transfusions.^39^, ^40^

### 3.1 History and timeline

**FIGURE 2:**
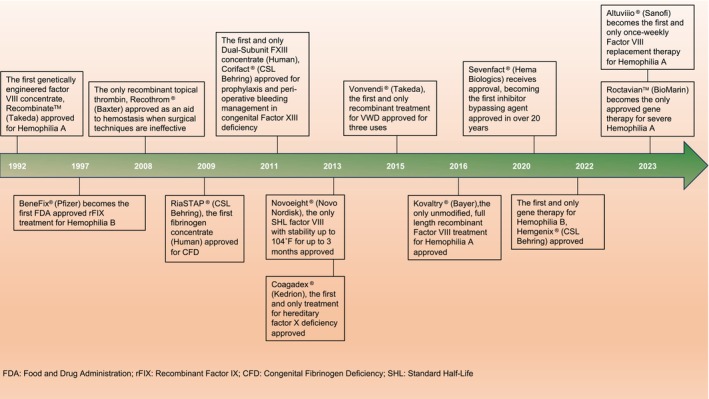
History and timeline of notable events in select FDA hemostat approvals.

The history of the evolution of commercial hemostats is depicted in Figure 2. Prior to the 1960s, the mainstay treatment of bleeding was whole blood and fresh frozen plasma.^13^ Cryoprecipitate for the treatment of hemophilia was introduced in the 1960, marking the first advancement in hemophilia therapies.^41^ In the 1970s, FVIII concentrates were manufactured from human plasma and proved to be effective for hemophilia treatment—but this success was short‐lived, since several patients contracted acquired immune deficiency syndrome (AIDS) that was transmitted by concentrates derived from pooled human plasma, leading to death tolls in the 1980s.^42^ To avoid the perils of bloodborne pathogens, heating as a method to inactivate the human immunodeficiency virus (HIV) from plasma‐derived concentrates gained significant impetus.^43^ In addition, the following decade witnessed the extensive efforts to develop recombinant protein‐based hemostats, which led to the approval of the first recombinant anti‐hemophilic factor, Recombinate™ in 1992, developed by Baxter. The year 1997 marked the next major milestone in the development of hemostats, when Pfizer's BeneFix®, the first recombinant Factor IX for the treatment of Hemophilia B was approved by the FDA.^44^

In addition to hemophilia, several therapies were also developed as an aid to hemostasis during surgical procedures and as a treatment option for other coagulation disorders. Recothrom®, approved in 2008, remains the only available recombinant topical thrombin used as an aid to hemostasis in surgeries. The year 2009 marked the approval of RiaSTAP®, the first fibrinogen concentrate designed to treat Congenital Fibrinogen Deficiency (CFD), a rare inherited bleeding disorder characterized by impaired blood clot formation.^45^ 2011 and 2013 witnessed the approval of two more clotting factors, a human FXIII concentrate, Corifact® and a human FX concentrate, Coagadex®. Notably, in 2013, Novoeight® (recombinant FVIII) with stability up to 104 °F for 3 months, as opposed to clotting factors with stringent refrigeration/freezing requirements, was approved. Over the next 5 years, recombinant vWF (Vonvendi®, 2015), unmodified recombinant FVIII (Kovaltry®, 2016), and an inhibitor‐bypassing agent (Sevenfact®, 2020), received FDA approval. Despite significant advances, absence of a cure remained an unmet need for Hemophilia patients. This was addressed by the development and subsequent approval of gene therapies, a milestone in hemophilia treatment. Hemgenix® (2022) and Roctavian™ (2023) were approved by the FDA for the treatment of Hemophilia B and severe Hemophilia A, respectively. Finally, the most recent approval of Altuviiio® in 2023 has introduced a Factor VIII with an extended plasma half‐life, enabling a once‐a‐week Factor VIII replacement therapy for Hemophilia A.^46^ Additionally, regulatory considerations for hemostatic agents have been addressed in other reviews.^47^, ^48^

### 3.2 Modality of approved hemostats

**FIGURE 3:**
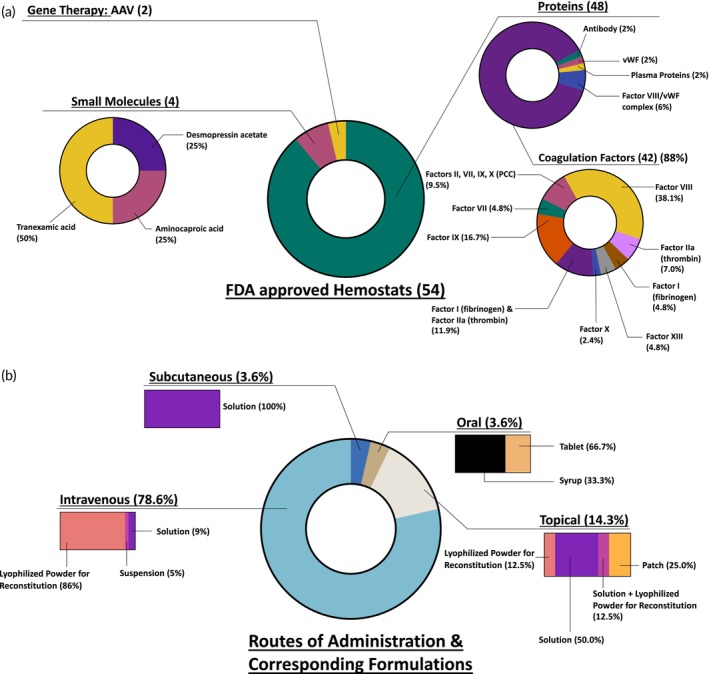
FDA approved hemostats. (a) Modality of approved hemostats. (b) Routes of administration and corresponding formulations of approved hemostats. AAV, adeno‐associated vector; vWF, von Willebrand factor.

The approved hemostat agents are primarily focused on three modalities including small molecules, proteins, and gene therapies (Figure 3a). Majority of the approved hemostats are protein‐based, encompassing 48 different products accounting for 89% of the total approved hemostatic agents. This trend aligns with the predominant focus in the field on developing human and recombinant coagulation factors, which collectively make up 88% of the approved proteins. Notably, this includes 16 Factor VIII products and 7 Factor IX products. Additionally, there are 4 approved Prothrombin Complex Concentrates (PCCs), featuring varying amounts of vitamin K‐dependent clotting factors and available as 3‐factor (Factors II, IX, X) or 4‐factor (Factors II, VII, IX, X) concentrates.^49^, ^50^ The FDA‐approved PCCs include Profilnine®, Feiba®, Kcentra® and Balfaxar®. Human plasma‐derived thrombin and fibrinogen are used as fibrin sealants in surgical settings. Currently approved fibrin sealants include Tisseel™, Evicel, Tachosil, Vistaseal™ and Evarrest. A detailed stratification of commercial products containing coagulation factors as the active ingredient and their mode of origin (human/recombinant) is shown in Table 2. This table also points to the various techniques employed to extend the half‐life of recombinant clotting factors, such as PEGylation, Fc fusion, and XTENylation.^51^, ^52^ Of note, Altuviiio® (Efanesoctocog alfa) is the most recently approved recombinant FVIII product with an extended half‐life. The active ingredient of Altuviiio is a Fc‐vWF‐XTEN fusion protein, which makes a once‐weekly factor VIII replacement therapy possible.^46^ In addition, the FVIII/vWF complexes including Alphanate®, Humate‐P® and Wilate® account for 6% of the approved protein‐based hemostats. Since a crucial physiological function of vWF is to act as a carrier for factor VIII to protect it from early proteolytic degradation, these complexes are used for the treatment of vWD and Hemophilia A as vWF reduces the immunogenicity of FVIII.^53^, ^54^, ^55^, ^56^, ^57^ Hemlibra® (Emicizumab) is the only bispecific antibody approved for the treatment of Hemophilia A, which works by restoring the function of the missing factor VIII (FVIII) by bridging FIXa and FX and therefore enabling hemostasis.^58^

**TABLE 2:** Commercially available human and recombinant coagulation factors/complexes.

Human coagulation factors/complexes		Recombinant coagulation factors/complexes			
Trade name	Active coagulation factor	Trade name	Active coagulation factor	Description	Producing cell line
Profilnine®	Coagulation Factors II, VII, IX and X	NovoSeven®	FVII	Active two‐chain form of FVIIa	BHK
FEIBA®	Coagulation Factors II, VII, IX and X	SevenFACT®	FVII	Activated coagulation FVII	Genetically engineered rabbits
Kcentra®	Coagulation Factors II, VII, IX and X, and anti‐thrombotic Proteins C and S	Recombinate™	FVIII	Full length FVIII	CHO
Balfaxar®	Coagulation factors II, VII, IX, and X and anti‐thrombotic Proteins C and S	ReFacto®	FVIII	BDD factor VIII	CHO
RiaSTAP®	FI	ADVATE®	FVIII	Full length FVIII	CHO
Fibryga®	FI	XYNTHA®	FVIII	BDD FVIII	CHO
AlphaNine®	FIX	Novoeight®	FVIII	BDT FVIII	CHO
KOATE®	FVIII	Obizur®	FVIII	BDD analogue of porcine FVIII	BHK
HemofilM®	FVIII	NUWIQ®	FVIII	BDD FVIII	HEK
Alphanate®	FVIII/vWF	KOVALTRY®	FVIII	Full length recombinant FVIII	BHK
HUMATE‐P®	FVIII/vWF	AFSTYLA®	FVIII	BDT FVIII, single chain	CHO
Wilate®	FVIII/vWF	ESPEROCT®	GlycoPEGylated FVIII	GlycoPEGylated, BDT FVIII	CHO
COAGADEX®	FX	ADYNOVATE®	PEGylated FVIII	PEGylated full‐length human coagulation FVIII	CHO
CORIFACT®	FXIII	JIVI®	PEGylated FVIII	PEGylated, BDD FVIII	BHK
TISSEEL™	Human plasma‐derived fibrinogen and thrombin	ELOCTATE®	FVIII‐Fc Fusion Protein	BDD FVIII, Fc fusion protein	HEK
Evicel®	Human plasma‐derived fibrinogen and thrombin	ALTUVIIIO®	FVIII‐Fc‐VWF‐(XTEN)2 FP	BDD FVIII covalently fused to IgG1 Fc domain, FVIII‐binding D'D3 domain of human vWF and 2 XTEN polypeptides	HEK
TachoSil®	Human plasma‐derived fibrinogen and thrombin	Tretten®	FXIII A‐Subunit	Homodimer composed of two FXIII A‐subunits	Saccharomyces cerevisiae
EVARREST®	Human plasma‐derived fibrinogen and thrombin	BeneFix®	FIX	Primary amino acid sequence identical to Ala148 allelic form of human FIX	CHO
Vistaseal™	Human plasma‐derived fibrinogen and thrombin	RIXUBIS®	FIX	Amino acid sequence identical to Ala‐148 allelic form of human FIX	CHO
Octaplas®	Plasma proteins	IXINITY®	FIX	Amino acid sequence comparable to Thr‐148 allelic form of human FIX	CHO
EVITHROM™	Thrombin	ALPROLIX®	FIX‐Fc FP	Human FIX sequence linked to IgG1 Fc domain; amino acid sequence identical to Thr148 allelic form of human FIX	HEK
THROMBIN‐JMI®	Thrombin^a^	IDELVION®	Albumin FP‐FIX	Human FIX sequence fused with recombinant albumin; amino acid sequence identical to Thr148 allelic form of human FIX	CHO
		Rebinyn®	GlycoPEGylated FIX	PEGylated, amino acid sequence identical to Thr148allelic form of human FIX	CHO
		RECOTHROM®	Thrombin	Full length human thrombin	CHO

Abbreviations: BDD, beta domain deleted; BDT, beta domain truncated; BHK, Baby Hamster Kidney; CHO, Chinese Hamster Ovary; FP, fusion protein; HEK, human embryonic kidney; IgG1, human immunoglobulin G1; vWF, von Willebrand Factor.

^a^ Thrombin JMI is sourced from bovine origin.

Four small molecules are approved as hemostats for various indications, making up 7.2% of all approved hemostats. Two of these (Lysteda® and Cyklokapron®) comprise of the antifibrinolytic agent, tranexamic acid. Tranexamic acid is a synthetic amino acid derivative of lysine, that reduces the degradation of hemostatic fibrin by the anti‐coagulant, plasmin. Tranexamic acid binds to the receptor binding sites on plasmin, thus inhibiting the binding of fibrin monomers to those sites. Since the fibrin monomers do not bind to the fibrinolytic plasmin, dissolution of plasmin is prevented and the structure of the fibrin matrix is retained.^59^, ^60^ Aminocaproic acid, marketed as Amicar®, is now available as generic equivalents. The fibrinolysis‐inhibitory effects of Amicar are exerted principally via the inhibition of plasminogen activators and through antiplasmin activity.^61^, ^62^ Lastly, desmopressin acetate, a vasopressin analog, increases the activity of Factor VIII in the plasma in patients with Hemophilia and Type 1 von Willebrand's disease.^63^

There are two gene therapies approved for the treatments of Hemophilias A and B. The first, etranacogene dezaparvovec (Hemgenix®) received FDA approval in 2022 and comprises of adeno‐associated virus (AAV)‐5 carrying a codon‐optimized DNA sequence of the gain‐of‐function Padua variant of human Factor IX, regulated by a liver‐specific promotor 1 (LP1), for the treatment of Hemophilia B. Subsequently, valoctocogene roxaparvovec (Roctavian™) received approval in 2023 for the treatment of severe Hemophilia A and consists of AAV‐5 carrying a DNA sequence of the B‐domain deleted SQ form of the human coagulation factor VIII (hFVIII‐SQ).^64^, ^65^

### 3.3 Route of administration and formulations

To assess the practicality of hemostat use, such as ease of administration, storage stability, and cold chain requirements, we analyzed the route of administration and formulations of the FDA approved products, depicted in Figure 3b. A notable 79% agents are administered intravenously, a logical choice given the need for immediate bioavailability in most prophylactic and bleeding treatment scenarios. The majority of intravenously injected agents are available as lyophilized powders for reconstitution, typically stored at 4°C with stability ranging from 12 to 36 months. Upon reconstitution, the solution must be administered promptly or within 3–4 h. Both gene therapies, Roctavian™ and Hemgenix®, are administered via intravenous infusion and formulated as suspensions. Hemgenix is stored in a refrigerator at 2–8°C, whereas Roctavian™ requires freezing at temperatures less than or equal to −60°C. Hemlibra®, a bispecific antibody, and desmopressin acetate, a vasopressin analog, are available as solutions stored at 4°C and administered subcutaneously. Oral formulations include the small molecules Amicar® (tablet and syrup) and Lysteda® (tablet). Commercially available thrombin products and fibrin sealants are applied topically at the bleeding site and their intravascular administration is strictly prohibited due to the risk of thrombosis. These come in various formulations, such as patches, solutions, and lyophilized powders, each with specific storage requirements.

### 3.4 Indications and active ingredients

**FIGURE 4:**
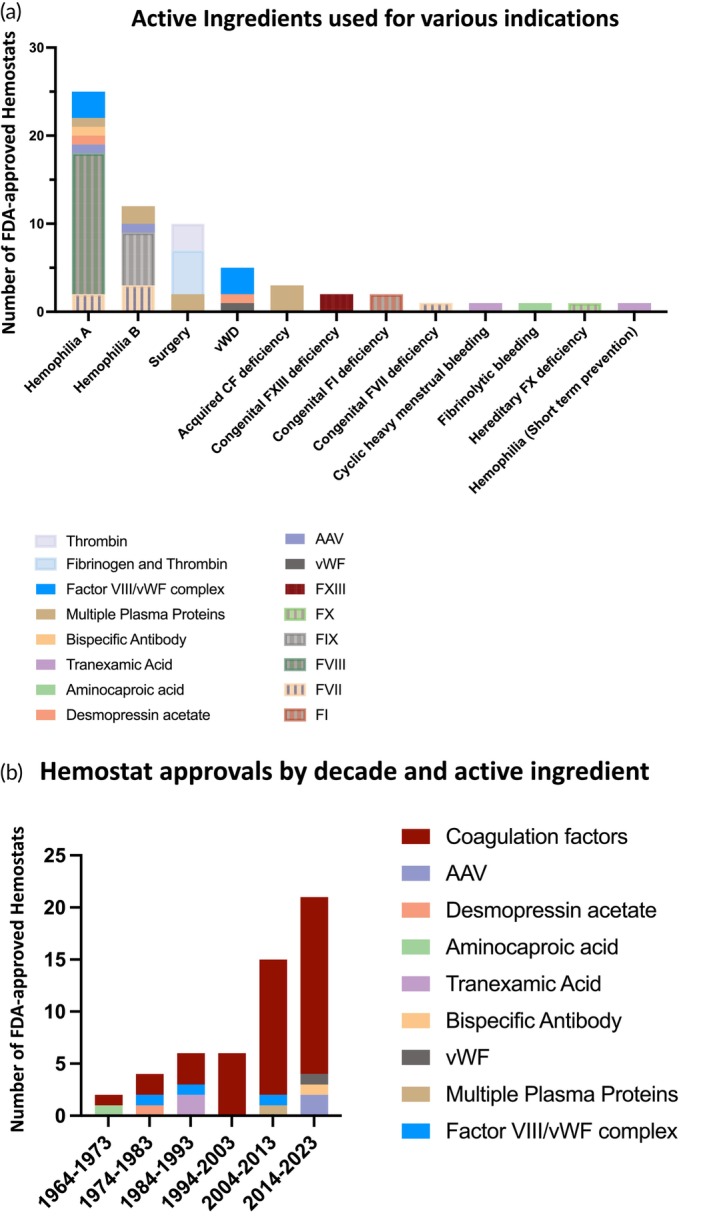
Distribution of indications and active ingredients, approval trends in FDA approved hemostats. (a) Number of hemostats approved per indication. (b) Number of hemostat approvals per decade. AAV, adeno‐associated virus; CF, coagulation factor; vWF, von Willebrand factor.

Based on the indications listed on their FDA approval labels, the 54 approved hemostats are used for the treatment of 12 unique indications, as shown in Figure 4a. In terms of the indications, majority of the products are for Hemophilias A and B, followed by surgery and vWD. Although the incidence of vWD is higher than that of hemophilia, the dominating indications of the approved products are the two types of hemophilia.^66^ Von Willebrand disease occurs as Type 1 (70%–80% cases, vWF deficiency), Type 2 (20% cases, dysfunctional vWF) or Type 3 (less than 5% cases, absence of circulating vWF).^17^ Agents approved for vWD treatment are specific for a particular type, for example, DDAVP® for Type 1 and Vonvendi® for Type 3. The remaining hemostats are used for the treatment of other coagulation factor deficiencies (FI, FX, FXIII) and specific types of bleeding, for example, Lysteda® (tranexamic acid) for cyclic heavy menstrual bleeding and Cyklokapron® (tranexamic acid) for short‐term prevention in hemophilia patients during and after tooth extraction. In terms of active ingredients, Hemophilia A has the widest array for prophylaxis/treatment. These ingredients include FVII, FVIII, FVIII/vWF complex, AAV, desmopressin acetate, bispecific antibody, and multiple plasma proteins (including PCC). The second widest array of actives is seen for Hemophilia B, which comprises of FVII, FIX, AAV, and multiple plasma proteins (including PCC). As shown in Figure 4b, the progress in the field of hemostats has seen steady growth from each decade to the next, along with the evolution of new active ingredients.

Of note, hemostats are utilized either for prophylaxis to prevent bleeding risks or for therapeutic purposes to control bleeding events, or for both. The various types of modalities offer a clear classification of the hemostat's final use. For example, the bispecific antibody Hemlibra® is intended for routine prophylaxis to prevent or reduce the frequency of bleeding episodes. Coagulation factor I products, thrombin products, fibrin sealants, and most small molecule products are exclusively used for the control of bleeding. Conversely, most standalone coagulation factors, such as Factors VII, FVIII, FIX and FX, are used for both prophylaxis and the control of bleeding conditions. Furthermore, the two most recent gene therapies, Roctavian® and Hemgenix®, are one‐time gene therapy products administered as a single dose via intravenous infusion for the treatment of Hemophilia A and B. A detailed description of the hemorrhage specifics, including use for prophylaxis or control of bleeding, can be found in Table 1.

### 3.5 Approved hemostats in active clinical trials

With numerous indications and subtypes within each indication for which hemostats are employed, ongoing clinical trials are investigating the utilization of previously approved hemostats for different conditions, as detailed in Table 3. The established safety and efficacy of approved therapeutics in humans, demonstrated through rigorous clinical trials, streamline the process of seeking approval for additional indications as compared to the development of entirely new therapeutics. Furthermore, if these approved therapeutics are commercialized, they are expected to adhere to rigorous standards of good manufacturing practice (GMP).

**TABLE 3:** FDA approved hemostats under active clinical trials for different indications.

Trade name	Approved indication	Investigated indication	Sponsor (collaborator)	NCT number [phase]
Balfaxar®	Acquired coagulation factor deficiencies	Acute Major Bleeding (on DOAC therapy with factor Xa inhibitor)	Octapharma^a^	NCT04867837 [3]
		Bleeding Cardiac Surgery Patients	Octapharma^a^	NCT05523297 [3]
Kcentra®	Acute major bleeding, surgery (acquired CF deficiency)	Traumatic Injury	Oregon Health and Science University	NCT04019015 [2]
		Traumatic Injury	CSL Behring^a^	NCT05568888 [3]
RiaSTAP®	Congenital fibrinogen deficiency	Scoliosis Surgery	Brno University Hospital	NCT05391412 [4]
		Trauma	Australian & New Zealand Intensive Care Research Centre	NCT05449834 [3]
Lysteda®	Cyclic heavy menstrual bleeding	Postoperative Blood Loss for hip fracture	Ascension Genesys Hospital	NCT05047133 [2/3]
Kovaltry®	Hemophilia A	Hemophilia A (Chinese children, adolescents/adults with severe Hemophilia A)	Bayer^a^	NCT04565236 [4]
Adynovate®	Hemophilia A	Hemophilia A (Chinese Men and Boys)	Takeda^a^	NCT05707351 [3]
		Hemophilia A (young children up to 5 years)	Takeda^a^	NCT02615691 [3]
Altuviiio®	Hemophilia A	Hemophilia A (previously treated with Altuviiio, newly initiated (in China only), prophylactic regimen ahead of major surgery	Sanofi^a^	NCT04644575 [3]
Roctavian™	Hemophilia A	Hemophilia A (Receiving Prophylactic Corticosteroids	BioMarin Pharmaceutical^a^	NCT04323098 [3]
		Hemophilia A (Receiving Prophylactic FVIII Infusions)	BioMarin Pharmaceutical^a^	NCT03370913 [3]
		Hemophilia A (with inhibitors to FVIII)	BioMarin Pharmaceutical^a^	NCT04684940 [1/2]
		Hemophilia A (with pre‐existing antibodies against AAV5)	BioMarin Pharmaceutical^a^	NCT03520712 [1/2]
		Severe Hemophilia A	BioMarin Pharmaceutical^a^	NCT02576795 [1/2]
Nuwiq®	Hemophilia A	Hemophilia A (women/girls undergoing major surgery)	Octapharma^a^	NCT05936580 [4]
	Hemophilia A	Severe Hemophilia A (undergoing major surgery while receiving emicizumab prophylaxis)	Octapharma^a^	NCT05935358 [4]
Hemlibra®	Hemophilia A	Hemophilia A (prophylaxis for patients who received episodic therapy with FVIII or bypassing agents prior to study)	Hoffmann‐La Roche	NCT03315455 [3]
		Mild Hemophilia A (Males, 5–45 years without inhibitors)	Indiana Hemophilia &Thrombosis Center, (Genentech^a^)	NCT04567511 [4]
		Mild or Moderate Hemophilia A (without FVIII inhibitors)	Hoffmann‐La Roche	NCT04158648 [3]
		Severe Hemophilia A (age 0–12 months)	Hoffmann‐La Roche	NCT04431726 [3]
		Von Willebrand Disease, Type 3|Concomitant VWD and Hemophilia	Bleeding and Clotting Disorders Institute Peoria, Illinois (Genentech^a^)	NCT05500807 [1]
		Acquired Hemophilia A	University of Washington (Genentech^a^)	NCT05345197 [2]
Esperoct®	Hemophilia A	Severe Hemophilia A (age 0–6 years); previously untreated	Novo Nordisk^a^	NCT02137850 [3]
Jivi®	Hemophilia A (ages 12 and older)	Hemophilia A (ages 7–12)	Bayer^a^	NCT05147662 [3]
Rebinyn®	Hemophilia B	Hemophilia B (previously treated children)	Novo Nordisk^a^	NCT01467427 [3]
		Hemophilia B (Chinese patients)	Novo Nordisk^a^	NCT05365217 [3]
Hemgenix®	Hemophilia B	Hemophilia B (to further describe safety profile)	CSL Behring^a^	NCT03569891 [3]
		Hemophilia B (with detectable pretreatment AAV5 Nabs)	CSL Behring^a^	NCT06003387 [3]
Feiba®	Hemophilia A and B	Hemophilia A (with inhibitors on emicizumab)	Children's Hospital Los Angeles (Takeda^a^)	NCT04205175 [4]
Novoseven®	Hemophilia A and B	Intracerebral Hemorrhage	University of Cincinnati, (Novo Nordisk^a^)	NCT03496883 [3]
Sevenfact®	Hemophilia A or B	Hemophilia A or B (with inhibitors in the presence or absence of prophylactic therapies)	American Thrombosis and Hemostasis Network	NCT04647227 [4]
Wilate®	Hemophilia A, vWD	Severe Bleeding (Given with platelet transfusions)	University Hospital, Basel	NCT04555785 [4]
		Severe Von Willebrand Disease (age 0–6 years)	Octapharma^a^	NCT04953884 [3]
Alphanate®	Hemophilia A, vWD (except type 3)	Von Willebrand Disease (Type 3)	Grifols Biologicals^a^	NCT00555555 [4]
Tisseel™	Surgery	Eyelid Surgery	University of Calgary	NCT05358977 [2/3]
Evarrest®	Surgery (adults)	Surgery (Pediatric Population)	Ethicon^a^	NCT03255174 [3]
Cyklokapron®	Tooth extraction hemorrhage	Gastric Bypass Hemorrhage	Franciscus Gasthuis	NCT05464394 [3]
		Intracerebral Hemorrhagic Stroke	Christian Medical College, Ludhiana	NCT05836831 [4]
		Open Cardiac Surgery	Population Health Research Institute	NCT03954314 [3]
		Postpartum Hemorrhage	Cairo University	NCT06010368 [3]
		Postpartum Hemorrhage	London School of Hygiene & Tropical Medicine	NCT05562609 [3]
		Postpartum Hemorrhage	George Washington University	NCT03287336 [2]
		Postpartum Hemorrhage	Karolinska Institutet	NCT06025916 [4]
		Sinus Surgery (Nebulized TXA)	Assiut University	NCT04905901 [3]
		Tangential Skin Excision	St. Olavs Hospital	NCT02918201 [2]
Vonvendi®	vWD (adults)	vWD (Pediatric and adult participants with severe vWD)	Takeda^a^	NCT03879135 [3]
		vWD (Children with severe vWD)	Takeda^a^	NCT05582993 [3]

Abbreviations: CF, coagulation factor; DOAC, direct oral anti‐coagulant; Nab, neutralizing antibody; TXA, tranexamic acid; vWD, von Willebrand Disease.

^a^ Company sponsoring/collaborating in the clinical trial is the same company holding the FDA approval for the original approved indication of the drug (as seen in Table 1).

Pfizer's Cyklokapron® stands out with the highest number of ongoing clinical trials. It was originally approved for tooth extraction hemorrhage but now under exploration for a diverse range of alternative hemorrhages such as postpartum hemorrhage, gastric bypass hemorrhage and intracerebral hemorrhagic stroke.^67^, ^68^, ^69^, ^70^, ^71^, ^72^, ^73^, ^74^, ^75^ The patient population nuances for Hemlibra® are under investigation in five distinct clinical trials, including one trial assessing its use for von Willebrand disease (vWD) instead of its original indication, Hemophilia A.^76^, ^77^, ^78^, ^79^, ^80^, ^81^ Similarly, the recently approved gene therapy Roctavian™ is further investigated in five ongoing clinical trials, all dedicated to studying its efficacy in specific Hemophilia A patient subpopulations. Notably, all trials have been sponsored by BioMarin, the original company securing Roctavian™'s FDA approval.^82^, ^83^, ^84^, ^85^, ^86^ Similarly, the recently approved gene therapy Hemgenix® is also being investigated in two clinical trials to further describe its safety profile and to explore its efficacy in patients with detectable neutralizing antibodies to the AAV serotype.^87^, ^88^ Alphanate®, approved in 1978 for Hemophilia and vWD (except Type 3), remains under an active clinical trial for vWD (Type 3), underscoring the enduring interest in exploring new indications for previously approved therapeutics.^89^

## 4 HEMOSTATS UNDER CURRENT CLINICAL TRIALS

Given the trend of an increasing number of hemostat approvals per decade, there is high interest in developing new agents as hemostats with better pharmacokinetic properties, improved efficacy and potential to treat newer indications. In this section, we overview the landscape of hemostatic agents under active clinical trials as of November 2023, identified from clinicaltrials.gov using the keywords “Hemostat OR Hemostasis OR Hemorrhage” that had the status “recruiting OR not yet recruiting OR active, not recruiting OR enrolling by invitation.” The retrieved entries (more than 550) underwent additional manual screening to selectively incorporate only those studies assessing investigational hemostats used as therapeutics. The complete list of 75 active clinical trials were identified and documented in Table 4.

**TABLE 4:** Hemostats in active clinical trials.

Generic name (code name^a^)	Condition	Active ingredient	Route	NCT number [phase]	Sponsor
Cell/gene therapy					
CD68‐ET3‐LV CD34+	Hemophilia A	Autologous HSCs: transduced with LV encoding FVIII	IV	NCT04418414 [1]	Expression Therapeutics
HSCT CD68‐ET3‐LV	Hemophilia A	Autologous HSCs: transduced with LV encoding FVIII	IV	NCT05265767 [1]	Christian Medical College, Vellore
(SPK‐8016)	Hemophilia A	AAV	IV	NCT03734588 [1/2]	Spark Therapeutics
Giroctocogene fitelparovec (PF‐07055480)	Hemophilia A	AAV 2/6 encoding FVIII	IV	NCT03061201 [2]	Pfizer
Giroctocogene fitelparovec (PF‐07055480)	Hemophilia A	AAV 2/6 encoding FVIII	IV	NCT04370054 [3]	Pfizer
(ZS802)	Hemophilia A	AAV encoding FVIII	IV	NCT05523128 [N/A]	Institute of Hematology & Blood Diseases Hospital, China
(GS001)	Hemophilia A	AAV encoding FVIII	IV	NCT04728841 [N/A]	Institute of Hematology & Blood Diseases Hospital, China
BAY2599023 (DTX201)	Hemophilia A	AAV encoding FVIII	IV	NCT03588299 [1/2]	Bayer
(ASC618)	Hemophilia A	AAV encoding FVIII	IV	NCT04676048 [1/2]	ASC Therapeutics
(AAV2/8‐HLP‐FVIII‐V3)	Hemophilia A	AAV8 encoding FVIII	IV	NCT03001830 [1]	University College, London
(BAX 888)	Hemophilia A	AAV8 encoding FVIII	IV	NCT03370172 [1/2]	Baxalta, Takeda
(SPK‐8011)	Hemophilia A	AV‐LK03 (Spark200) encoding FVII	IV	NCT03003533 [1/2]	Spark Therapeutics
(VGB‐R04)	Hemophilia B	AAV encoding FIX	IV	NCT05152732 [1^b^]	Institute of Hematology & Blood Diseases Hospital, China
(BBM‐H901)	Hemophilia B	AAV encoding FIX	IV	NCT04135300 [N/A]	Institute of Hematology & Blood Diseases Hospital, China
(ZS801)	Hemophilia B	AAV encoding FIX	IV	NCT05630651 [N/A]	Institute of Hematology & Blood Diseases Hospital, China
(BBM‐H901)	Hemophilia B	AAV encoding FIX	IV	NCT05709288 [1]	Institute of Hematology & Blood Diseases Hospital, China
(VGB‐R04)	Hemophilia B	AAV encoding FIX	IV	NCT05441553 [1/2]	Shanghai Vitalgen BioPharma Co.
(ZS801)	Hemophilia B	AAV encoding FIX	IV	NCT05641610 [1/2]	Institute of Hematology & Blood Diseases Hospital, China
Fidanacogene elaparvovec (PF‐06838435 (formerly SPK‐9001))	Hemophilia B	AAV encoding FIX	IV	NCT03307980 [2]	Pfizer
Fidanacogene elaparvovec (PF‐06838435 (formerly SPK‐9001))	Hemophilia B	AAV encoding FIX	IV	NCT03861273 [3]	Pfizer
(BBM‐H901)	Hemophilia B	AAV encoding FIX	IV	NCT05203679 [3]	Shanghai Belief‐Delivery BioMed Co.
scAAV2/8‐LP1‐hFIXco	Hemophilia B	AAV2/8 encoding FIX	IV	NCT00979238 [1]	St. Jude Children's Research Hospital
AskBio009 (BAX 335)	Hemophilia B	AAV8 encoding FIX	IV	NCT01687608 [1/2]	Takeda
Fitusiran	Hemophilia A and B	siRNA	IV	NCT03754790 [3]	Sanofi
Fitusiran	Hemophilia A and B	siRNA	IV	NCT05662319 [3]	Sanofi
Miscellaneous					
Chinese herbal medicine (FYTF‐919)	ICH	Miscellaneous (herbs)	Oral	NCT05066620 [3]	Guangzhou University of Traditional Chinese Medicine
Arista	Surgery	Miscellaneous (plant starch)	Topical	NCT05522153 [1]	Virtua Health
PuraBond	Surgery	Miscellaneous (Peptides self‐assembling into ECM mimicking scaffold)	N/A	NCT05773781 [N/A]	University of Liverpool
Proteins					
NNC0365‐3769 (Mim8)	Hemophilia A	FVIII mimetic bispecific antibody	SC	NCT05306418 [3]	Novo Nordisk
NNC0365‐3769 (Mim8)	Hemophilia A	FVIII mimetic bispecific antibody	SC	NCT05053139 [3]	Novo Nordisk
NNC0365‐3769 (Mim8)	Hemophilia A	FVIII mimetic bispecific antibody	SC	NCT05685238 [3]	Novo Nordisk
NNC0365‐3769 (Mim8)	Hemophilia A	FVIII mimetic bispecific antibody	SC	NCT05878938 [3]	Novo Nordisk
(NXT007)	Hemophilia A	FVIII mimetic bispecific antibody	SC	NCT05987449 [1/2]	Hoffmann‐La Roche
Concizumab	Hemophilia A and B	Anti‐TFPI monoclonal antibody	SC	NCT05135559 [3]	Novo Nordisk
Concizumab	Hemophilia A and B	Anti‐TFPI monoclonal antibody	SC	NCT04083781 [3]	Novo Nordisk
Concizumab	Hemophilia A and B	Anti‐TFPI monoclonal antibody	SC	NCT04082429 [3]	Novo Nordisk
(MG1113)	Hemophilia A and B	Anti‐TFPI monoclonal antibody	SC	NCT05493631 [1]	GC Biopharma
(KN057)	Hemophilia A and B	Anti‐TFPI monoclonal antibody	SC	NCT05421429 [2]	Suzhou Alphamab
Marstacimab (PF‐06741086)	Hemophilia A and B	Anti‐TFPI monoclonal antibody	SC	NCT03938792 [3]	Pfizer
Marstacimab (PF‐06741086)	Hemophilia A and B	Anti‐TFPI monoclonal antibody	SC	NCT05145127 [3]	Pfizer
Marstacimab (PF‐06741086)	Hemophilia A and B	Anti‐TFPI monoclonal antibody	SC	NCT05611801 [3]	Pfizer
(TU7710)	Hemophilia A and B	Bypassing agent	IV	NCT06025552 [1]	TiumBio
(SS109)	Hemophilia A and B	Factor VIIa‐Fc Fusion Protein	IV	NCT06010953 [1/2]	Jiangsu Gensciences
Recombinant FVIIa	Hemophilia A and B	FVII	IV	NCT05487976 [3]	Chia Tai Tianqing Pharmaceutical Group
Recombinant Factor VIIa (LR769)	Hemophilia A and B	FVIIa	IV	NCT05695391 [3]	Laboratoire francais de Fractionnement et de Biotechnologies
(STSP‐0601)	Hemophilia A and B	FX activator	IV	NCT05619926 [2]	Staidson Biopharmaceuticals
SerpinPC	Hemophilia A and B	Novel inhibitor of APC	IV or SC	NCT04073498 [1/2]	ApcinteX Ltd
SerpinPC	Hemophilia A and B	Novel inhibitor of APC	SC	NCT05789524 [2]	ApcinteX Ltd
SerpinPC	Hemophilia B	Novel inhibitor of APC	SC	NCT05789537 [2]	ApcinteX Ltd
Fibrinogen	Major Hemorrhage	Fibrinogen (FI)	IV	NCT05300672 [3]	Azienda Usl di Bologna
Fibrinogen concentrate human	Major Hemorrhage	Fibrinogen (FI)	IV	NCT05091684 [2]	Centre Hospitalier Universitaire de Saint Etienne
Bentracimab (PB2452)	Major Hemorrhage	Monoclonal antibody	IV	NCT04286438 [3]	PhaseBio Pharmaceuticals
4 factor prothrombin complex concentrates	Major Hemorrhage	PCC	IV	NCT05738642 [N/A]	Second Affiliated Hospital, Zhejiang University
sFilm‐FS	Surgery	Fibrin Sealant	Topical	NCT04660721 [1/2]	Sealantium Medical
(BT524)	Surgery	Fibrinogen	IV	NCT03444324 [3]	Biotest
(TAK‐330)	Surgery	PCC	IV	NCT05156983 [3]	Takeda
Small molecules					
Tranexamic acid	Antepartum hemorrhage	TXA	IV, oral	NCT05840471 [N/A]	Hawler Medical University
Tranexamic acid	ICH	TXA	IV	NCT04742205 [1^b^]	Kathmandu Medical College and Teaching Hospital
Terlipressin	Major Hemorrhage	Terlipressin	IV	NCT06027970 [3]	Postgraduate Institute of Medical Education and Research
Tranexamic acid	Major Hemorrhage	TXA	IV	NCT04387305 [3]	Daniel Nishijima, University of California, Davis
Tranexamic acid	Major Hemorrhage	TXA	IM	NCT04521881 [3]	London School of Hygiene and Tropical Medicine
Tranexamic acid	Major Hemorrhage	TXA	IV	NCT05053867 [3]	M.D. Anderson Cancer Center
Tranexamic acid	Postpartum Hemorrhage	TXA	IV	NCT03069859 [2]	Sunnybrook Health Sciences Centre
Tranexamic acid	Postpartum Hemorrhage	TXA	IV	NCT05370820 [2]	George Washington University
Tranexamic acid	Postpartum Hemorrhage	TXA	IV	NCT05811676 [3]	Guangzhou Medical University
Tranexamic acid	Postpartum Hemorrhage	TXA	IV	NCT05759156 [N/A]	Dow University of Health Sciences
Tranexamic acid	Postpartum Hemorrhage	TXA	Topical	NCT05072873 [N/A]	Aswan University Hospital
Tranexamic acid	Postpartum Hemorrhage	TXA	IV	NCT04304625 [3]	University Hospital, Bordeaux
Tranexamic acid	Surgery	TXA	N/A	NCT05507983 [3]	Maasstad Hospital
Tranexamic acid	Surgery	TXA	IV	NCT04311073 [3]	Eastern Virginia Medical School
Tranexamic acid	Surgery	TXA	IV	NCT05427513 [1^b^]	Shaukat Khanum Memorial Cancer Hospital & Research Centre
Tranexamic acid	Surgery	TXA	IV	NCT05230381 [N/A]	Beijing Tiantan Hospital
Tranexamic acid	Surgery	TXA	SC	NCT06057675 [2]	Vanderbilt University Medical Center
Tranexamic acid	Surgery	TXA	IV	NCT05774717 [1]	Vanderbilt University Medical Center
Tranexamic acid	Surgery	TXA	Topical	NCT05152186 [3]	Minia University

Abbreviations: AAV, adeno‐associated virus; APC, activated protein C; ECM, extra‐cellular matrix; HSC, hematopoietic stem cells; ICH, intracerebral hemorrhage; LV, lentiviral vectors; PCC, prothrombin complex concentrate; SC, subcutaneous; siRNA, small interfering RNA; TFPI, tissue‐factor pathway inhibitor; TXA, tranexamic acid.

^a^ Code name mentioned wherever applicable.

^b^ Early Phase 1 trial.

As noted earlier with the FDA‐approved hemostats, advancements are also occurring in transfusion medicine, particularly in the realm of platelet transfusions. While cold‐stored platelets were previously employed in clinical settings, they fell out of favor due to their quicker clearance from circulation, and hence, room‐temperature stored platelets were preferred.^90^ However, there is a renewed interest in investigating cold‐stored platelets, particularly in surgical settings. Cold storage offers advantages such as limiting bacterial growth, enhancing hemostatic efficacy, and eliminating the agitation requirement during storage, as seen with their room‐temperature counterparts.^91^, ^92^, ^93^ Clinical trials are currently exploring the utilization of cold‐stored platelets in cardiac surgery and trauma, showcasing a revived interest in their potential benefits.^94^, ^95^, ^96^, ^97^ Moreover, ongoing trials are investigating the use of deep‐frozen platelets and whole blood for surgery and trauma, introducing innovative approaches to transfusion strategies.^98^, ^99^, ^100^, ^101^

In addition to cold‐stored and deep‐frozen platelets, lyophilized platelets have emerged as hemostatic agents, offering the distinct advantage of a prolonged shelf life of up to 3 years at ambient temperatures. Leading lyophilized platelet products include Stasix® and Thrombosomes®, which utilize paraformaldehyde and trehalose, respectively, to stabilize platelet membranes during lyophilization and rehydration.^102^ Stasix® has been evaluated in swine bleeding models but is not currently under investigation in clinical trials.^103^ On the other hand, Thrombosomes®, developed by Cellphire Therapeutics,^104^ showed promising results in Phase 1 trials^105^, ^106^; however, the Phase 2 study in thrombocytopenic patients was terminated due to funding issues.^107^ Presently, Thrombosomes® are undergoing a Phase 2 trial for use as a hemostat in acute thoracic aortic dissections.^108^ The diverse forms of platelets—cold‐stored, deep‐frozen and lyophilized—represent exciting avenues for future explorations.^109^

To address some of the logistical challenges associated with the use of Fresh Frozen Plasma (FFP), an alternative plasma formulation, freeze‐dried plasma (FDP), has been explored.^110^ Freeze‐dried plasma, manufactured by freeze‐drying a large batch of plasma units, can be stored at room temperature for up to 2 years and easily reconstituted with sterile water for injection.^111^ Although the use of FDP is not new, several issues related to disease transmission have hindered its wide adoption.^112^ However, with improvements in donor screening, testing procedures, and pathogen reduction technology, FDP is emerging as a potential hemostatic agent, especially since the manufacturing of the French military's lyophilized plasma.^113^ Although extensive clinical trial data on the safety and efficacy of FDP compared to standard‐care fresh plasma is not available, the results of a few clinical trials using FDP as a hemostat provide interesting insights.^114^, ^115^, ^116^, ^117^, ^118^

### 4.1 Modalities of hemostats in clinical trials

**FIGURE 5:**
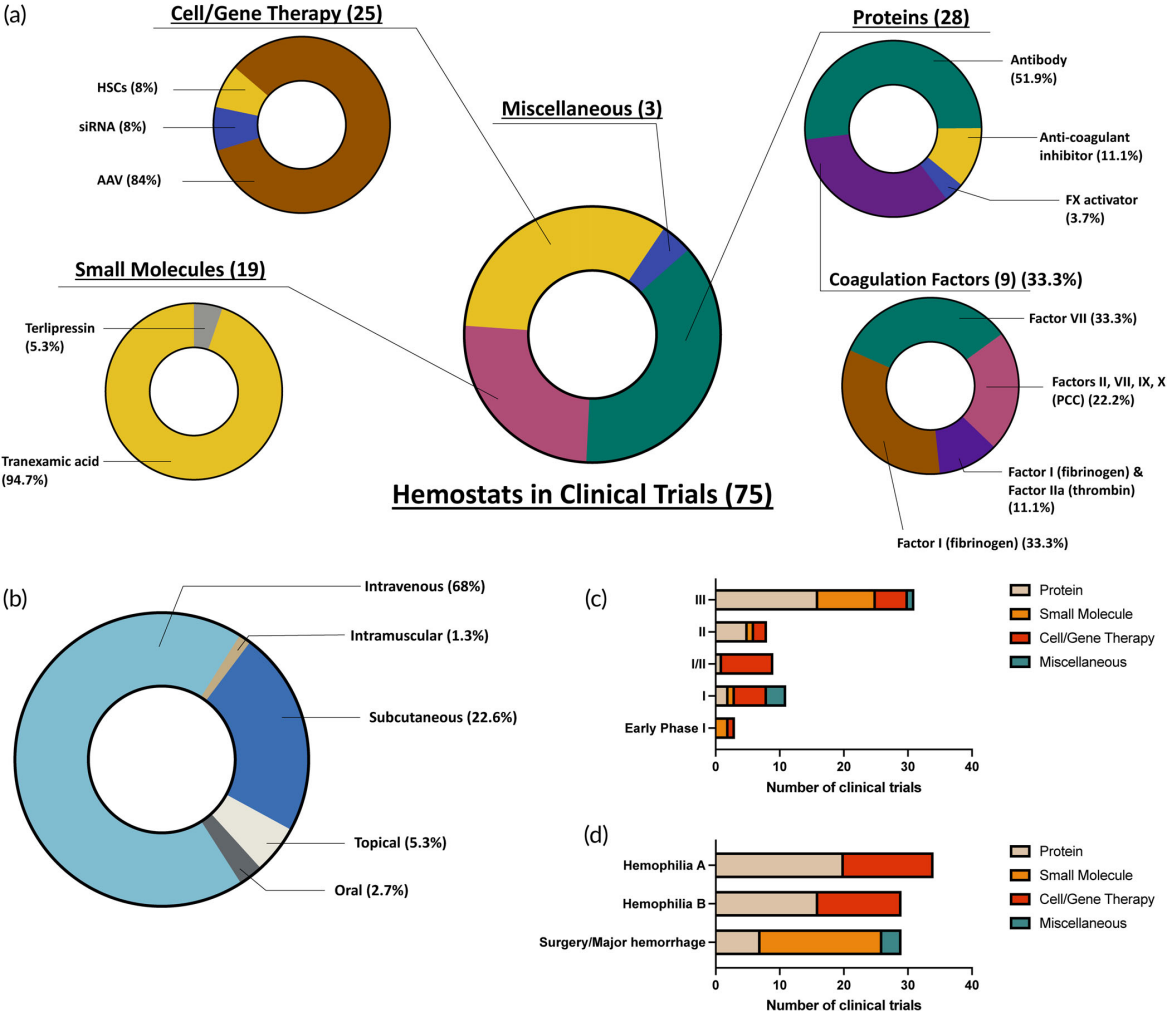
Hemostats in clinical trials. (a) Modality of investigational hemostats. (b) Route of administration of investigational hemostats. (c) Number of clinical trials of hemostats per indication. (d) Number of clinical trials of hemostats per phase. AAV, adeno‐associated virus; HSCs, hematopoietic stem cells; siRNA, small interfering RNA; vWD, von Willebrand disease. vWF, von Willebrand factor.

As illustrated in Figure 5a, the two primary modalities, accounting for 37.3% and 33.3% of the active clinical trials, are proteins and cell/gene therapy agents, respectively. Protein‐based hemostats, while not as dominant as in FDA‐approved products, still make up a sizeable portion of active clinical trials and constitute four main protein classes. Of these, notably, antibodies emerge as a noteworthy focus in clinical trials, showcasing an interesting shift in the major proteins under investigation. Of these, NXT007 and Mim8 are both bispecific antibodies to FIXa and FX, similar to the only approved hemostatic antibody, Hemlibra.^119^, ^120^, ^121^, ^122^, ^123^, ^124^, ^125^ There are eight ongoing trials for the candidates Concizumab, Marstacimab, KN057, MG1113, and NXT007, all of which are monoclonal antibodies acting as anti‐tissue factor pathway inhibitors (TFPI). Since TFPI regulates the extrinsic pathway of coagulation by inhibiting FXa and extrinsic FXase, neutralizing TFPI increases the extrinsic pathway activity, thus promoting hemostasis.^126^, ^127^, ^128^ The second most dominant protein subtype used in the active hemostat‐focused clinical trials is coagulation factors. Interestingly, there are currently no new FVIII, FIX or vWF agents undergoing clinical trials for bleeding indications. There is a noticeable increase in FI and FVII candidates under investigation. A novel inhibitor of endogenous anti‐coagulant activities, specifically the activated protein C (APC), is being investigated in the trial of the candidate SerpinPC. A snake venom derived Factor X activator, STSP‐0601, is another protein being studied for the treatment of hemophilia.^129^, ^130^

Cell and Gene therapy agents constitute the second most prevalent modality of hemostat agents in clinical trials, constituting 25 of the identified ongoing trials. The increasing number of these trials, coupled with the recent approvals for gene therapy, serves as an indicator of the evolving landscape in the field. A significant proportion of these trials focus on AAV‐based therapy for Hemophilia A and B. Beyond AAV5 which is the serotype used in the two FDA‐approved AAV‐based products for hemophilia, exploration also extends to other serotypes such as AAV 2/8, AAV8, and a chimeric capsid (AAVLK03). Additionally, two siRNA‐focused trials involving the product Fitusiran are underway for hemophilia treatment. Fitusiran, an siRNA directed against anti‐thrombin, has demonstrated a dose‐dependent reduction in serum anti‐thrombin levels in pre‐clinical models.^131^ Finally, two ongoing trials explore the use of hematopoietic stem cells (HSCs) transduced with a lentiviral vector encoding a Factor VIII transgene for the treatment of Hemophilia A.

Small molecule based hemostats, constituting 25.3% of the identified clinical trials, are primarily represented by tranexamic acid, with one ongoing clinical trial for Terlipressin, a vasopressin analog. Three agents fall under the category of “miscellaneous” either because they do not precisely align with previously mentioned modalities or due to insufficient available data regarding their composition. These miscellaneous agents include a Chinese herbal medicine (FYTF‐919) intended for the treatment of intracerebral hemorrhage (ICH), PuraBond—a self‐assembling peptide hydrogel designed for use in surgical settings—and Arista, which contains plant starch and is to be employed in surgical applications.

### 4.2 Route of administration and type of formulation

Similar to the trend observed for the FDA approved products, the primary route of administration used for the investigative hemostats under active clinical trials is the intravenous route, accounting for 68% of the identified trials, as shown in Figure 5b. All gene therapy and recombinant protein‐based hemostats are administered intravenously. The second most prevalent route of administration is the subcutaneous route, used in 22.6% of the identified trials. All antibodies under investigation, along with one tranexamic acid preparation and the APC inhibitor, SerpinPC, are administered subcutaneously. The only intramuscular injection is observed in a clinical trial exploring the use of tranexamic acid for the treatment of Traumatic Brain Injury.^132^ Further, 5.3% of the investigative hemostat agents use topical application as the route of administration. This includes a fibrin sealant, sFilm‐FS, a patch embedded with lyophilized powders of human fibrinogen, human thrombin and calcium chloride for use in hepatic surgery.^133^ Lastly, two candidates—one involving tranexamic acid (TXA) and the other rooted in Chinese traditional medicine—are administered orally.

### 4.3 Clinical trial phases

Figure 5c shows the distribution of modalities as per the phase of clinical trials. Three agents are in early phase I trials, which involve exploratory trials conducted before traditional phase 1 to investigate the effect of the drug on the body. These agents include one AAV gene therapy and two small molecules. The nine candidates undergoing Phase 1 trials cover all four major modalities—small molecules, proteins, gene therapy and miscellaneous. Two of these explore the use of HSCs transduced with a lentiviral vector for the treatment of Hemophilia A. Another candidate is TU7710 (Tium Bio), a bypassing agent for patients with neutralizing antibodies to Hemophilia drugs with a greater half‐life than conventional hemophilia treatments.^134^ Notably, 12 candidates are indicated for both Phase 1 and Phase 2. These include eight AAV therapeutics, such as SPK‐8016 and SPK‐8011 (Spark Therapeutics), DTX‐201(Bayer), ASC618 (ASC Therapeutics), BAX‐888 (Baxalta, Takeda) for the treatment of Hemophilia A, and VGB‐R04 (Shanghai Vitalgen BioPharma), ZS‐801 (Institute of Hematology & Blood Diseases Hospital, China) and BAX‐335 (Baxalta, Takeda) for the treatment of Hemophilia B. Nine candidates undergoing Phase 2 trials include 2 of Pfizer's gene therapy candidates, Giroctocogene fitelparovec (PF‐07055480) and Fidanacogene elaparvovec (PF‐06838435; formerly SPK‐9001) for the treatments of Hemophilias A and B, respectively. Two of the three SerpinPC protein trials (ApcinteX, Centessa Pharmaceuticals) are among the five proteins undergoing Phase 2 trials. The largest number of candidates are seen in Phase 3, where 16 proteins, 9 small molecules, 1 miscellaneous and 5 gene therapy agents constitute a total of 31 active ongoing clinical trials. A notable gene therapy candidate in Phase 3 is Sanofi's siRNA candidate, Fitusiran for hemophilia. Notable protein candidates in Phase 3 trials include Novo Nordisk's FVIII mimetic bispecific antibody, Mim8 and anti–tissue factor pathway inhibitor monoclonal antibody, concizumab, along with Pfizer's anti–tissue factor pathway inhibitor monoclonal antibody, marstacimab (PF‐06741086).

### 4.4 Investigated indications

The indications for agents under active clinical trials can be broadly classified into three main categories: Hemophilia A, Hemophilia B and Surgery/major hemorrhage as shown in Figure 5d. Hemophilia A is the most sought‐after indication, accounting for 37% of the ongoing clinical trials. This is followed by Hemophilia B and surgery/major hemorrhage, accounting for roughly 32% each. For the two types of hemophilia, the modality is approximately an even split between gene therapy and proteins, with 55% proteins and 45% gene therapy agents for Hemophilia B, and 59% proteins and 41% gene therapy agents for Hemophilia A. These ratios, compared to the approved modalities for both hemophilia types, point to the rising interest in gene therapy agents as a treatment option. Surgeries and major hemorrhages are the other major indication observed in the investigative hemostats in active clinical trials. Hemorrhages including antepartum hemorrhage, postpartum hemorrhage, ICH, variceal hemorrhage and surgeries including spine surgery, burn excision surgery and hepatic surgery are included in this category. Notably, 65% of the agents investigated for the surgery and major hemorrhage indications are based on small molecule such as TXA and Terlipressin. In addition, 24% of the agents are based on proteins, such as Phase Bio's monoclonal antibody (Bentracimab, aka PB2452) for the reversal of anti‐platelet effect,^135^, ^136^ Takeda's four‐factor prothrombin complex concentrate (TAK‐330) for the reversal of factor Xa‐induced anti‐coagulation,^137^ and Biotest's Human Fibrinogen Concentrate (BT524) for major blood loss during elective spine surgery.^138^

## 5 CASE STUDY: HEMOPHILIA A AND HEMOPHILIA B

**FIGURE 6:**
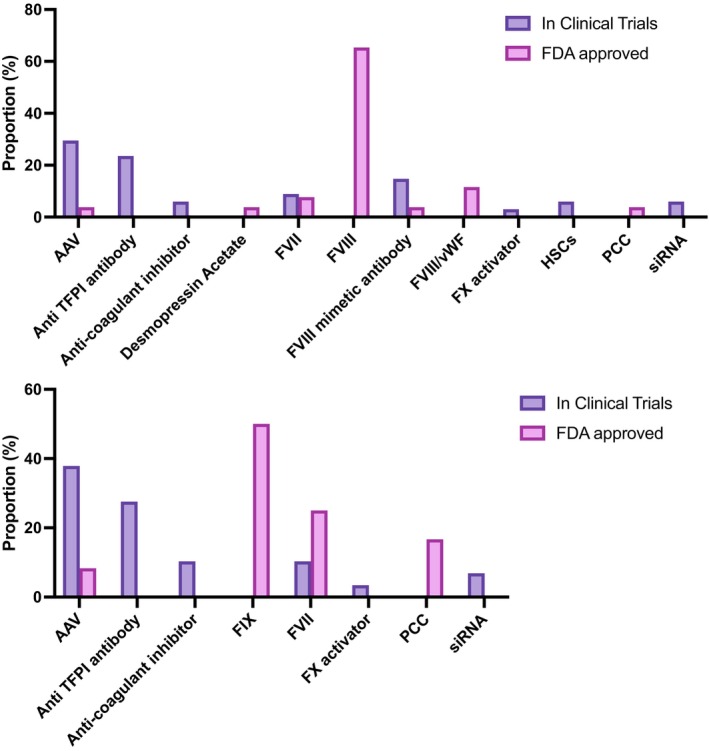
Stratification of active ingredients in clinical trials and FDA approved hemostats for Hemophilia A and B. (a) Proportion of active ingredients for Hemophilia A. (b) Proportion of active ingredients for Hemophilia B. AAV, adeno‐associated virus; HSCs, hematopoietic stem cells; PCC, Prothrombin Complex Concentrate; siRNA, small interfering RNA. TFPI, Tissue Factor Pathway Inhibitor; vWF, von Willebrand factor.

Considering the prevalence of Hemophilia A and B as indications observed in both the FDA‐approved and investigative hemostats, we perform a further analysis of the active ingredients used in the hemostatic agents to address Hemophilia. Specifically, we conduct a comparative analysis of the active ingredients used in the FDA‐approved products versus those used in investigative products, aiming to highlight some emerging trends in the treatment options for this coagulation disorder (Figure 6).

For Hemophilia A, Factor VIII replacement therapy dominates in the FDA approved products, accounting for 64% of all approved active ingredients. Notable examples of such Factor VIII concentrates include Koate® (human), HemofilM® (human), Recombinate™ (recombinant), Altuviiio (recombinant). This is followed by FVIII/vWF and FVII products, along with FVIII mimetic antibody, PCC, DDAVP® and AAV. A significantly distinct landscape is observed for agents in active clinical trials for Hemophilia A. The largest proportion of active ingredients in the investigative hemostats under active clinical trials is AAV therapy, followed closely by anti‐TFPI antibodies and FVIII mimetic antibodies. Additional novel active agents such as siRNA, HSCs, anti‐coagulant inhibitor and FX activator, for which there are no corresponding approved products, are also seen in clinical trials.

While the spectrum of active ingredients used in the hemostat agents for Hemophilia B is not as broad as that for Hemophilia A, we still observed some defining trends when comparing the approved and investigative products. For the FDA‐approved agents, FIX is the most dominant ingredient, used in 50% of the approved products such as in BeneFix® (recombinant) and Rebinyn® (recombinant). This is then followed by FVII, PCC, and AAV. In comparison, we do not observe novel FIX and PCC products being explored in clinical trials for the treatment of Hemophilia B. As with Hemophilia A, AAV therapy makes up the highest proportion, with several clinical trials for AAVs encoding novel FIX transgenes underway, such as Takeda's BAX‐335 and Pfizer's PF‐06838435.^139^, ^140^, ^141^ This is then followed by anti‐TFPI antibodies, anti‐coagulant inhibitors and FVII along with novel agents such as siRNA and FX activator.

## 6 CONCLUSION

Given their vital role in both emergency and prophylactic settings, hemostats remain indispensable tools in addressing heavy bleeding and coagulation complications. Thousands of hemostat devices have received approvals from regulatory agencies and are utilized in emergency situations, while numerous others serve as therapeutics in clinical settings. Ongoing clinical trials are actively investigating a diverse range of hemostats as therapeutic products, spanning from traditional protein‐based to new modalities such as gene therapy. In response to the evolving landscape, efforts are underway to enhance the usability and administration of these agents. Continuous innovations in design also aim to extend the shelf‐life and simplify the storage requirement of hemostats. These developments are crucial in addressing a wide array of coagulation disorders and surgical bleeds, with indications expanding to cover various medical scenarios. The increasing number of hemostat approvals per decade serves as a testament to the continual evolution of the field, marked by innovations in every aspect of the therapy. Clinical trials are exploring novel active ingredients to target common conditions, paving the way for potentially more efficacious therapies as the hemostat field enters the seventh decade since the approval of the first product.

In addition, whether a hemostat is donor‐derived or donor‐independent significantly influences drug development and associated risks. Donor derived products, such as platelets and plasma‐derived coagulation factors, face challenges including limited availability, variable product quality, limited shelf life, immunologic reactions, and the risk of bacterial contamination and disease transmission. There are, however, opportunities to address these challenges with donor‐derived products. On the other hand, donor‐independent modalities, such as small molecule hemostats, recombinant coagulation factors, and gene therapies, while addressing the limitations of donor‐derived hemostats, encounter their own unique set of drug development challenges. For example, concerns about the long‐term outcomes of tranexamic acid, a small molecule used as an antifibrinolytic in menstrual bleeding and hemophilia, have been raised.^142^

Regarding commercially available coagulation factors and complexes, there is nearly an equal number of products derived from human sources and those produced recombinantly. The development of neutralizing antibodies against coagulation factors, known as inhibitors, is a crucial aspect of assessing the efficacy of coagulation factor replacement products. For instance, the source of FVIII products—whether plasma‐derived or recombinant—remains one of the most significant factors impacting inhibitor development. Recent epidemiological studies have documented differential immunogenicity of these concentrates depending on their source, although the underlying causes remain poorly understood.^143^, ^144^

Gene therapy for hemophilia represents a promising one‐time cure, leading to the sustained endogenous production of FVIII or FIX proteins at levels sufficient to restore normal hemostasis. This approach mitigates the risks associated with nonadherence to frequent factor replacement therapy. However, uncertainties remain, hindering the transition from factor replacement proteins to gene therapy. These uncertainties include the identification of suitable candidates for gene therapy, the unpredictability of response, long‐term efficacy and safety concerns, and the potential need for immunosuppression.^145^

Looking ahead, it is crucial to monitor the advancements in innovative protein products and the growing trend toward gene therapy, which holds the potential to offer more effective therapies for bleeding disorders. Additionally, exploring the pre‐clinical and clinical development of agents addressing internal bleeding in non‐compressible hemorrhages resulting from trauma, while mitigating the risk of thromboembolic complications, present an unmet avenue for investigation. The evolution of hemostats is anticipated to benefit from new advances in multidisciplinary fields such as in protein engineering and genetic engineering. These technological advancements are expected to facilitate significant strides in the field, fostering innovation and providing solutions for a wide range of hematological conditions.

## AUTHOR CONTRIBUTIONS

**Maithili Joshi:** Conceptualization; formal analysis; methodology; validation; writing – original draft; writing – review and editing. **Zongmin Zhao:** Conceptualization; writing – review and editing. **Samir Mitragotri:** Conceptualization; funding acquisition; supervision; writing – review and editing.

## CONFLICT OF INTEREST STATEMENT

SM is an inventor on patent applications in the field of hemostats (owned and managed by Harvard University).

### PEER REVIEW

The peer review history for this article is available at https://www.webofscience.com/api/gateway/wos/peer‐review/10.1002/btm2.10673.

## Data Availability

All data are available in the main manuscript or supplementary materials.
